# Stem cell systems and regeneration in planaria

**DOI:** 10.1007/s00427-012-0426-4

**Published:** 2012-11-09

**Authors:** Jochen C. Rink

**Affiliations:** Max Planck Institute of Molecular Cell Biology and Genetics, Pfotenhauerstrasse 108, 01307 Dresden, Germany

**Keywords:** Planaria, Stem cells, Pluripotency, Regeneration, Homeostasis

## Abstract

Planarians are members of the Platyhelminthes (flatworms). These animals have evolved a remarkable stem cell system. A single pluripotent adult stem cell type (“neoblast”) gives rise to the entire range of cell types and organs in the planarian body plan, including a brain, digestive-, excretory-, sensory- and reproductive systems. Neoblasts are abundantly present throughout the mesenchyme and divide continuously. The resulting stream of progenitors and turnover of differentiated cells drive the rapid self-renewal of the entire animal within a matter of weeks. Planarians grow and literally de-grow (“shrink”) by the food supply-dependent adjustment of organismal turnover rates, scaling body plan proportions over as much as a 50-fold size range. Their dynamic body architecture further allows astonishing regenerative abilities, including the regeneration of complete and perfectly proportioned animals even from tiny tissue remnants. Planarians as an experimental system, therefore, provide unique opportunities for addressing a spectrum of current problems in stem cell research, including the evolutionary conservation of pluripotency, the dynamic organization of differentiation lineages and the mechanisms underlying organismal stem cell homeostasis. The first part of this review focuses on the molecular biology of neoblasts as pluripotent stem cells. The second part examines the fascinating mechanistic and conceptual challenges posed by a stem cell system that epitomizes a universal design principle of biological systems: the dynamic steady state.

## Primer: what are planarians?

Planarians are flat, soft-bodied worms with a triploblastic body plan and lacking visible segmentation. Many hundred species are known, including fresh- and seawater dwellers and even terrestrial forms. They are members of the phylum Platyhelminthes (platy = flat; helminth = worm), which additionally includes the parasitic clades Cestoda (tapeworms), Trematoda (flukes) and Monogenea (fish gill parasites). “Planaria” is a colloquial term that generally refers to free-living members of the order Tricladida. Planarians have long since attracted the attention of biologists due to their astonishing regenerative abilities, food supply dependent scaling of body size and their great abundance of adult stem cells. The fresh water species are easy and cheap to maintain in the laboratory and several species are studied. The two “workhorses” are *Schmidtea mediterranea* (*Smed*) and *Dugesia japonica* (*Dj*). Both have excellent regenerative abilities and clonal strains originating from single animals are used. Whether a particular research group works on *S*. *mediterranea* or *D*. *japonica* is mainly a question of habit and results are so far assumed to be comparable. Other planarian model species include *Schmidtea polychroa* (*Spol*; used as embryogenesis model due to consistent fecundity) and *Dugesia ryukyuensis* (*Dr*; model for switching between sexual and asexual reproduction modes) (Ishizuka et al. 2007). Gene names in the planarian literature carry a prefix designating the species (Reddien et al. 2008) (e.g., *Smed*-*actin* for *Schmidtea mediterranea* actin). The genome of *S*. *mediterranea* has been sequenced (Robb et al. 2008), and a genome project for *D*. *japonica* is underway. The current planarian tool kit further includes organism-wide RNAi (Sánchez Alvarado and Newmark 1999; Reddien et al. 2005a), BrdU-labeling (Newmark and Sánchez Alvarado 2000), in situ hybridization (Pearson et al. 2009; Umesono et al. 1997), FACS fractionation of stem cell populations (Hayashi and Agata 2012; Hayashi et al. 2006) and next generation sequencing techniques (Friedländer et al. 2009; Palakodeti et al. 2008). Beyond the Tricladidans, the flatworm species *Macrostomum lignano* is increasingly studied (Morris et al. 2006). Flatworms are amongst the first model systems within the so far scarcely investigated superphylum Lophotrochozoa and therefore also provide interesting evolutionary perspectives.

## Introduction

**Fig. 1:**
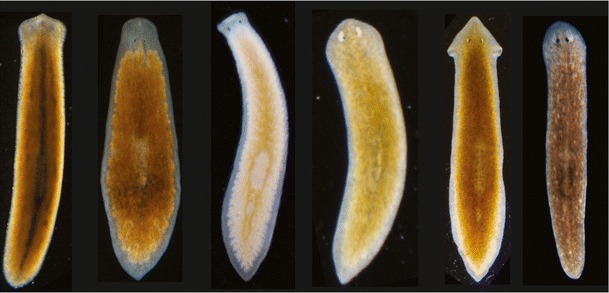
Examples of European planarian species. From left to right: *Polycelis* sp., *Planaria torva* (recently fed), *Dendrocoelum lacteum*, *Schmidtea polychroa*, *Dugesia gonocephala* (recently fed), *Schmidtea mediterranea*

Superficially, fresh water planarians may seem rather boring — flattened, mostly drab-colored worms without visible appendages (Fig. 1). A closer look reveals a set of organ systems similar to other triploblastic animals: A brain comprising diverse neurotransmitter systems (Umesono and Agata 2009), a highly branched gastrovascular cavity tasked with both the digestion and distribution of nutrients (Forsthoefel et al. 2011), a protonephridial excretory system with interesting evolutionary homologies to the vertebrate kidney (Rink et al. 2011; Scimone et al. 2011), diverse suites of sensory organs and a hermaphroditic reproductive system (Newmark et al. 2008). However, planarians are truly astonishing in terms of their biology. Akin to mythological beasts, they have the ability to regenerate in their entirety even from tiny injury remnants and the asexual strains appear to be exempt from the mortal’s plight of physiological ageing (Mouton et al. 2011; Pearson and Sánchez Alvarado 2008; Tan et al. 2012).

Both traits originate from an abundance of adult stem cells. Collectively referred to as “Neoblasts”, these cells have been investigated for more than a century (Baguñà 2012). Recently, Wagner and colleagues showed that transplantation of a single Neoblast into a lethally irradiated (=stem cell depleted) worm rescued the recipient and gave rise to a perfectly healthy animal of the donor genotype (Wagner et al. 2011). This experiment conclusively demonstrated the *pluripotency* of Neoblasts (defined here as the ability to differentiate into all zygotic cell types). Neoblasts are likely even *totipotent* (differentiation into all zygotic cell types + extraembryonic tissues), but the use of donor Neoblasts from asexual animals in the above experiments precluded the reconstitution of sexual reproduction and thus the formal demonstration of totipotency. Neoblasts as naturally occurring pluripotent adult stem cells are remarkable, because adult stem cells in other model systems are lineage-restricted and somatic pluripotency exists only transiently during early embryonic development. A further unusual feature of planarian Neoblasts is their high basal mitotic activity. The resulting stream of progenitors drives the continuous turnover of all planarian tissues, which are likely devoid of any long-lived cell types. Dynamic turnover characterizes also the vertebrate intestine, for example (van der Flier and Clevers 2009). Yet the complete turnover of an entire triploblastic animal in a matter of weeks is surely a further fascinating feature of planarians.

The above points raise a number of intriguing questions with respect to planarian biology and stem cell systems in general: What makes Neoblasts pluripotent and is pluripotency evolutionarily conserved? Which principles and mechanisms orchestrate the orderly generation of all organismal cell types from one pluripotent stem cell population? What maintains organismal homeostasis in face of changing flux rates between stem- and differentiated cell compartments? The revival of planarians as molecular model system is starting to provide insights into the above questions. This text takes a deliberately stem cell focused approach. Several recent reviews examine in detail regeneration, pattern formation or the unusual embryonic development of planarians (Tanaka and Reddien 2011; Forsthoefel and Newmark 2009; Aboobaker 2011; Adell et al. 2010; Martín-Durán et al. 2012; Reddien 2011).

## “Neoblasts”: definitions and lack thereof

The long history of Neoblast research has been recently reviewed (Baguñà 2012). Nevertheless, the lasting legacy of the pre-molecular era requires a brief historical excursion also at this point. The term “Neoblast” came into use to describe the small, roundish cells found abundantly throughout the planarian mesenchyme, except for the area in front of the photoreceptors and the pharynx (which are the only areas incapable of regeneration) (Reddien and Alvarado 2004). In transmission electron microscope (TEM) images, Neoblasts appear as 5- to 10-μm diameter cells with a thin rim of cytosol, lots of free ribosomes, few discernible organelles, prominent chromatoid bodies (CBs) (see below) and a large nucleus with little heterochromatin (Pedersen 1959; Hori 1982; Hay and Coward 1975; Coward 1974). The accumulation of Neoblasts at regenerating wounds and their rapid loss after regeneration-inhibiting doses of irradiation linked Neoblasts to regeneration (Wolff and Dubois 1948). The observation that all cell divisions in planarians occurred exclusively in cells meeting the above morphological criteria culminated in the statement “in planarians, Neoblasts are the only cells that divide” (Morita and Best 1984). This made the term “Neoblasts” practically synonymous with “dividing cells”. Consequently, generic cell division markers such as phospho-Histone H3 Ser10 (H3P), BrdU incorporation, or expression of cell division machinery components such as *PCNA* (Newmark and Sánchez Alvarado 2000; Orii et al. 2005; Salvetti et al. 2000) have been and are commonly used as Neoblast markers.

*PCNA* and H3P as stem cell markers? In other systems, true stem cells capable of self-renewal and the production of multiple progeny are generally rare amongst the cycling cells within a tissue (Weissman 2000). Transit amplifying cells, that is dividing stem cell progeny with limited self-renewal potential, tend to constitute the greatest proportion of mitotic cells and even differentiated cell types like fibroblasts or endothelial cells may divide occasionally in vertebrates. Generic cell division markers such as *PCNA* are consequently of little use in singling out stem cells from other dividing cell types. In planarians, however, cell divisions never occur within the confines of differentiated tissues. Not even high turnover tissues like gut or epidermis harbor dividing cells (Forsthoefel et al. 2011; Baguñà 1976a; Newmark and Sánchez Alvarado 2000), contrasting sharply with the high rates of cell divisions and organ-specific stem cell populations in the corresponding tissues of vertebrates or the *Drosophila* gut (van der Flier and Clevers 2009; Blanpain and Fuchs 2009; Micchelli and Perrimon 2006). Instead, planarian cell divisions are strictly limited to the loosely organized mesenchyme surrounding all organs and, more specifically, to cells meeting the morphological definitions of Neoblasts detailed above. Stating that “Neoblasts are the only dividing cells in planarians” therefore accurately captures the restriction of mitotic activity to one morphologically homogenous cell population and the absence of organ-specific stem cells or autonomously self-renewing cells outside the planarian mesenchyme.

On the other hand, the term “Neoblasts” and general statements in the planarian literature about what Neoblasts do or do not do often imply functional homogeneity in absence of hard evidence. For example, it is simply not known whether all PCNA- or H3P-positive “Neoblasts” are bonafide stem cells or whether planarians also harbor transient amplifying cell types that would consequently contribute to the PCNA-positive population. Moreover, neither morphological criteria nor the current molecular markers can draw an exact boundary between dividing cells and early committed progeny (see below). It is therefore important to stress that the term “Neoblasts” in the planarian literature and explicitly also in this text needs to be understood as a general reference to the planarian stem cell system, inclusive of pluripotent stem cells, earliest postmitotic progeny and any possible intermediate stages. The source of such ambiguity is not the use of a historical term, but the current lack of knowledge regarding the planarian stem cell system. Instead of introducing new terminology, I will therefore adapt the term “cNeoblasts” (“clonogenic Neoblasts”) from Wagner et al. for explicit references to pluripotent stem cells (Wagner et al. 2011) and “Neoblast progeny” when referring to early postmitotic differentiation stages. Neoblast heterogeneity will be further discussed below.

## The hunt for Neoblast genes

**Fig. 2:**
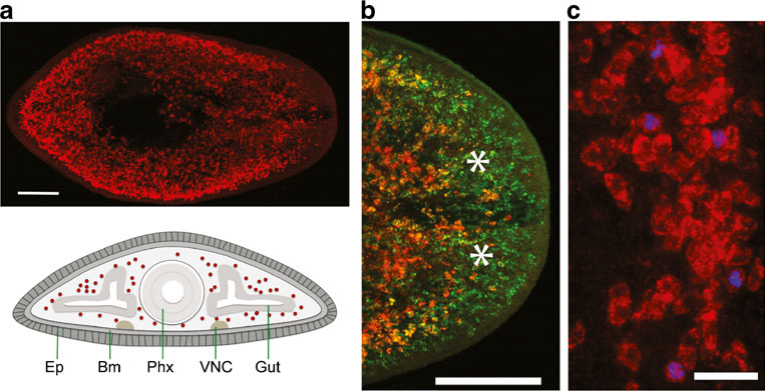
Organization of the planarian stem cell system. **a** *Top*: stem cell distribution as visualized by whole mount in situ hybridization with the neoblast marker *smedwi*-*1*. The image is a maximum intensity projection spanning the D/V axis (anterior is to the right). The stem cell devoid pharynx occupies the dark central area. Scale bar: 200 μm. *Bottom*: cartoon illustration of the stem cell distribution in a transverse section at the level of the pharynx. Stem cells (*red*), *Ep* epithelium, *Bm* basement membrane, *Phx* pharynx, *VNC* ventral nerve cords, *Gut* lateral branch of the gastrovascular system. **b** Magnified head region. *smedwi*-*1* RNA expressing Neoblasts (*red*) are largely absent from the area in front of the photoreceptors (*asterisks*), but SMEDWI-1 protein persists in anteriorly migrating stem cell progeny (*green*, antibody staining). Scale bar: 200 μm. **c** Only *smedwi*-*1* RNA expressing cells (*red*) divide (*blue*, H3P antibody staining). Scale bar: 25 μm

Pluripotent stem cells as potentially unlimited in vitro source of each and every cell type are of the greatest interest to regenerative medicine. Adult vertebrates do not harbor pluripotent stem cells, but pluripotent cells can be derived either from early embryos (embryonic stem [ES] cells; Weissman 2000) or via the recently discovered “reprogramming” of somatic cells back into a quasi-embryonic state (induced pluripotent stem [IPS] cells; Takahashi and Yamanaka 2006). Fundamental questions remain regarding the mechanistic basis of cellular pluripotency and the orderly transition to progenitor differentiation. Their continuous activity, abundance in adult tissues and evolutionary distance to vertebrates predestine planarian Neoblasts as model system for stem cell pluripotency (Fig. 2).

Understanding the mechanistic basis of Neoblast pluripotency consequently has been and continues to be a major focus of planarian research. The operational definition of Neoblasts as only dividing cells and the rapid elimination of dividing cells by X-irradiation have strongly shaped the experimental approaches. Comparisons between irradiated and non-irradiated samples yielded riboprobes for the localization of Neoblasts (Shibata et al. 1999; Salvetti et al. 2000; Reddien et al. 2005b), gating criteria for isolating Neoblast-containing cell populations by FACS (Hayashi et al. 2006) and, more recently, a basis for large-scale surveys of radiation-sensitive gene expression profiles (Rossi et al. 2007; Eisenhoffer et al. 2008; Wagner et al. 2012; Solana et al. 2012; Friedländer et al. 2009; Blythe et al. 2010). Further, the stereotypical progression of head regression, ventral curling and eventual lysis following lethal irradiation (Bardeen and Baetjer 1904) established a benchmark for Neoblast ablation RNAi phenotypes (Reddien et al. 2005a, b). Recently, Neoblast depletion by RNAi against a Neoblast specific Histone variant H2B has been proposed as more specific alternative to irradiation, circumventing the substantial transcriptional responses to organism-wide DNA damage (Solana et al. 2012).

Amongst the long list of genes now known to be expressed in and/or required for Neoblast function, three groups clearly stand out (Solana et al. 2012; Wagner et al. 2012; Labbé et al. 2012; Eisenhoffer et al. 2008; Rossi et al. 2007; Shibata et al. 2012; Onal et al. 2012; Rouhana et al. 2010): First, and hardly surprising given the experimental premises, components of the cell division machinery. Telomerase is found in this category and its regulation entails intriguing responsiveness to regeneration and reproduction modes (Tan et al. 2012). Second, a striking number of evolutionarily conserved proteins involved in posttranscriptional regulation of gene expression. Third and barely characterized, a growing list of chromatin modifiers.

## Piwi homologues as Neoblast markers

The planarian *piwi* homologues have arguably received the greatest attention amongst Neoblast markers. *S*. *mediterranea piwi*-*1* (*smedwi*-*1*) is highly expressed in an abundant cell population between the gut branches (Fig. 2a). *smedwi*-*1* ^+^ cells are absent from the pharynx and the area in front of the photoreceptors (Fig. 2b), become almost undetectable within 24 h of irradiation (Reddien et al. 2005b) and only *smedwi*-*1* expressing cells divide (Fig. 2c) (Eisenhoffer et al. 2008; Guo et al. 2006; Yoshida-Kashikawa et al. 2007). The additional *S*. *mediterranea piwi* homologues -2 and -3 are likewise expressed in Neoblasts (Reddien et al. 2005b; Palakodeti et al. 2008; Nakagawa et al. 2012b). Further, RNAi-mediated knock down of smedwi-2 and -3 or the respective *Dugesia ryukyuensis* homologues result in lethal Neoblast depletion, recapitulating the stereotypical deterioration of irradiated animals (Reddien et al. 2005b; Palakodeti et al. 2008; Nakagawa et al. 2012b). Overall, the planarian *piwi* genes therefore extend the morphological definition of Neoblasts to the molecular level.

Piwi and the closely related Argonautes are highly conserved mediators of gene regulation (Farazi et al. 2008). Argonautes are widely expressed and mediate gene silencing via small RNA like miRNA and siRNAs. In contrast, piwi homologues in established model systems are mostly germ line specific. The planarian *piwi* gene family has undergone a drastic expansion, generating at least seven *piwi* genes in *S*. *mediterranea* as opposed to three family members in fly or mouse (Juliano et al. 2011; Palakodeti et al. 2008; Friedländer et al. 2009). One essential function of piwi proteins is transposon silencing via piwi-associated small RNAs (piRNA) (Thomson and Lin 2009), sparking the honorific “guardians of the genome” (O’Donnell and Boeke 2007). Transposons have been estimated to account for 31% of the *S*. *mediterranea* genome (Friedländer et al. 2009). Since transposon-mediated mutations and genome rearrangements are likely as important a concern in indefinitely self-renewing pluripotent Neoblasts as in the germ line, could the essential role of *piwi* genes in Neoblasts reflect a similar role in the maintenance of genome integrity?

At least indirect evidence for a role of planarian PIWI proteins in transposon silencing comes from a recent study investigating the evolutionarily conserved arginine methyl-transferase Smed-PRMT-5 (Rouhana et al. 2012). PRMT-5 methylates RG containing motifs in RNA-binding proteins such as PIWI, SmB and Vasa, which subsequently serve as docking sites for proteins containing methyl-binding TUDOR domains. This interaction network provides an evolutionarily conserved assembly scaffold for ribonucleoprotein (RNP) complexes such as germ granules. Germ granules are a near-universal feature of germ cells and play essential roles in their formation and maintenance (see below) (Voronina et al. 2011; Eddy 1975). The conspicuous CBs of planarian Neoblasts are also RNPs (Auladell et al. 1993; Hori 1982; Coward 1974) and Rouhana et al. found that CBs carry SMED-PRMT-5 methylations. Importantly, *smedwi*-*3* was identified as PRMT-5 substrate and *PRMT*-*5*(*RNAi*) resulted in gradual Neoblast depletion and eventual death of the animals. The concomitant and massive upregulation of transposon transcription and the enrichment of piRNA-like RNA in CBs demonstrated a function of CBs and *smedwi*-*3* in transposon silencing (Rouhana et al. 2012). Consistently, abundant and diverse populations of likely piRNAs have been identified in Neoblasts, of which >30% map to annotated transposons (Palakodeti et al. 2008; Friedländer et al. 2009). However, the rapid decline of Neoblast numbers after *smedwi*-*3*(*RNAi*) and especially *smedwi*-*2*(*RNAi*) in comparison to the gradual decline after *PRMT*-*5*(*RNAi*) strongly suggests that the planarian PIWI proteins are required for more than transposon silencing, as are their germ line counterparts in other organisms (Klenov et al. 2011; Juliano et al. 2011). In fact, the *smedwi*-*2* and -*3*(*RNAi*) phenotypes suggest a role in Neoblast differentiation as well as self-renewal (Reddien et al. 2005b; Wagner et al. 2012). The future characterization of PIWI-associated small RNAs might identify the relevant targets and thus provide mechanistic insights into maintenance and exit from pluripotency.

## Chromatoid bodies and germ granules

Besides *smedwi*-*3* and *PRMT*-*5*, a surprising number of known Neoblast genes have links to CBs. The TUDOR homologue SPOL-TUD localizes to CBs and *Spol*-*TUD*-*1*(*RNAi*) depletes Neoblasts with very similar kinetics to *Smed*-*PRMT*-*5*(*RNAi*), consistent with a scaffold function of methyl-RG/TUDOR interactions in CBs (Solana et al. 2009; Rouhana et al. 2012). However, neither RNAi treatment completely disassembled CBs, which could indicate redundant mechanisms or molecular heterogeneity amongst CBs. The DEAD-box RNA helicase DJ-CBC-1 is required for Neoblast differentiation (Rouhana et al. 2010) and localizes to a subset of CBs, consistent with the latter possibility (Yoshida-Kashikawa et al. 2007). SMED-SmB, a further likely substrate of PRMT-5 (Rouhana et al. 2012), localizes to CBs and *SmB*(*RNAi*) causes rapid chromatoid body disassembly and Neoblast loss within a matter of days (Fernandéz-Taboada et al. 2010). RNAi against the RNA binding proteins *Smed*-*bruli* (a Bruno homologue) (Guo et al. 2006), *Dj*-*pumilio* (Salvetti et al. 2005) and *Smed*-*vasa*-*1* (Wagner et al. 2012) all result in the loss of Neoblasts and/or differentiation defects.

The above list of molecules is intriguing also because it reads like a veritable “who is who” in germ line specification. Homologues of the above neoblast constituents are generally necessary for germ granule and/or germ cell function, revealing molecular parallels between CBs and germ granules and between Neoblasts and germ cells in general (Shibata et al. 2010; Wang et al. 2010; Wagner et al. 2012; Solana et al. 2012; Voronina et al. 2011; Ewen-Campen et al. 2010).

## Neoblasts and germ cells

Parallels between germ cells and Neoblasts are surprising, because Neoblast are not germ cells. Sexually reproducing planarians harbor a bonafide germ line as part of their hermaphroditic reproductive system (Newmark et al. 2008). Both germ line and the somatic gonads can regenerate *de novo*, likely involving a population of putative germ line stem cells. These cells are very similar to “ordinary” Neoblasts in terms of morphology and radiation sensitivity, even though they may cycle at a slower rate (Wang et al. 2007; Sato et al. 2006; Handberg-Thorsager and Saló 2007). Interestingly, the factor that distinguishes somatic Neoblasts from germ line Neoblasts is expression of *nanos*, so far the only highly conserved germ line determinant that is not constitutively expressed in all Neoblasts (Wang et al. 2007; Sato et al. 2006; Handberg-Thorsager and Saló 2007). Consistently, *nanos*(*RNAi*) does not affect Neoblast maintenance, but prevents gonad regeneration (Wang et al. 2007; Nakagawa et al. 2012a). Neoblasts therefore appear poised fascinatingly close to the soma/germ line divide, requiring only a small push involving *nanos* expression to transition into germ line fate. The resemblance between Neoblasts and germ cells could reflect a peculiarity of planarian biology. However, the pluripotency of Neoblasts provides a conceptual link between the two cell types. Even though germ cells only give rise to gametes, gamete fusion during fertilization initiates development of the complete organism, thus necessitating pluripotency within the germ line. The ectopic differentiation of somatic cell types in germ line tumors (Teratomas) dramatically emphasizes the cryptic pluripotency of germ line cells (Juliano et al. 2010; Ciosk 2006; Seydoux and Braun 2006). Could the striking component sharing between germ cells and Neoblasts at the level of their RNPs reflect an ancient role of these structures in pluripotency (Juliano et al. 2010; Seydoux and Braun 2006)?

“Germ granules” are likely a heterogenous collection of germ line RNPs, which share components with RNPs in somatic cells (Anderson and Kedersha 2009). CB-like RNPs also occur in planarian neurons (Yoshida-Kashikawa et al. 2007) and the commonly observed expression of “Neoblast genes” in neurons might reflect similar component sharing (Solana et al. 2009, 2012; Rouhana et al. 2010; Rossi et al. 2012; Wagner et al. 2012). Germ granule disassembly frequently results in germ line loss and induction of excess granules can induce ectopic germ cells (reviewed by Ewen-Campen et al. 2010). Such importance of RNPs arises from their role as veritable control centers of posttranscriptional gene regulation (PTGR), including the transcriptional repression and storage of mRNA, transcript degradation via piRNA and miRNA, translational activation of specific RNAs and even epigenetic chromatin modification (reviewed by Voronina et al. 2011). Germ granules therefore likely exert a considerable influence on the translatable pool of mRNAs and thus ultimately the proteome of the cell. This role might be of particular importance to pluripotent cells: Their ability to differentiate into all cell types necessitates globally accessible chromatin, yet at the same time the emergence of differentiation promoting transcriptional circuits must be suppressed (reviewed by Koh et al. 2010). Pluripotency therefore might generally come at the cost of reduced transcriptional control options, consistent with widespread low-level transcription of the genome in pluripotent ES cells (Efroni et al. 2008; Gaspar-Maia et al. 2011). PTGR via germ granules and CBs could supply additional layers of gene expression control important for stabilizing the pluripotent state (Seydoux and Braun 2006; Juliano et al. 2010). Further, the predominance of PTGR in the orchestration of germ cell development has been interpreted in favor of reduced transcriptional control options (Ewen-Campen et al. 2010). PTGR components are also strikingly enriched in Neoblasts (Rouhana et al. 2010; Solana et al. 2012; Onal et al. 2012; Labbé et al. 2012), including the practically Neoblast-specific expression of what common knowledge would consider generic translation initiation factors (Rouhana et al. 2010). PTGR can therefore be expected to play similarly important roles during Neoblast maintenance and differentiation as during germ line differentiation, possibly reflecting common pluripotency related constraints on transcriptional control mechanisms.

## Transcriptional circuits in pluripotency

However, the discovery that overexpressing a cocktail of transcription factors can convert terminally differentiated cells back into a pluripotent state has placed dramatic emphasis on the importance of transcriptional mechanisms in the establishment and maintenance of pluripotency (Takahashi and Yamanaka 2006). IPS cell formation usually requires expression of the POU-domain transcription factor Oct4 and Sox2, which via mutual binding sites in their promoters establish self-maintaining transcriptional networks incorporating the pluripotency factor Nanog (Ng and Surani 2011). However, only a small fraction of cells overexpressing all reprogramming factors ever become pluripotent (Takahashi and Yamanaka 2006). Chromatin rearrangements are thought to constitute a rate limiting step in pluripotency establishment (Rouhana et al. 2012; Koh et al. 2010). Epigenetic chromatin modifications as well as the mechanistic contributions of Oct4/Sox2/Nanog targets to the pluripotent state are therefore an intense focus of current stem cell research.

Which, if any, of the above mechanisms participate in Neoblast pluripotency? Besides the much-reduced content of heterochromatin in TEM images (Hay and Coward 1975), practically nothing is known about chromatin regulation in planarian Neoblasts. The striking enrichment of chromatin modifiers in Neoblast gene expression profiles (Solana et al. 2012; Wagner et al. 2012; Onal et al. 2012; Labbé et al. 2012) emphasize the urgent need for detailed studies. A planarian Nanog-homologue has not been reported yet, but Neoblasts specifically express Sox- and POU-domain transcription factors (Wagner et al. 2012; Onal et al. 2012) and *Smed*-*soxP1* is required for Neoblast maintenance (Wagner et al. 2012). Further research needs to address whether these factors maintain a molecularly conserved pluripotency network or whether they carry out general “housekeeping” functions in planarian Neoblasts. Two recent studies directly compared Neoblasts to vertebrate stem cells. Onal et al. examined statistically the relative homologue abundance of vertebrate pluripotency factors and known Oct4/Nanog target genes amongst Neoblast transcriptomes and proteomes (Onal et al. 2012). In both cases, the authors found statistically significant enrichments, consistent with broad evolutionary conservation of pluripotency. Similarly, Labbé and colleagues reported particularly high degrees of overlap between a vertebrate pluripotency associated gene set and genes specifically upregulated in Neoblasts (Labbé et al. 2012). Together, these findings provide an exciting starting point for the comparative dissection of pluripotency across large evolutionary distances (planaria: protostomes/lophotrochozoa; vertebrates: deuterostomes). A first requirement are data addressing the function of planarian Sox and POU factors in molecular detail, including DNA binding sites, regulatory targets and cross-species rescue experiments in vertebrates. Should such studies reveal the existence of an ancient pluripotency core network, comparisons between Neoblasts and ES/IPS cells could identify conserved components/targets and thus aide in the mechanistic understanding of pluripotency. The alternative outcome, namely pluripotency as an epiphenomenon not relying on explicitly conserved molecular circuits, would be equally interesting. In this case, comparisons could help in revealing the general cellular processes that collectively give rise to pluripotency as an emergent systems property (e.g., “open chromatin”, “PTGR”, particular metabolic states). Further, the mechanistic contribution of CBs and germ granules to pluripotency remain an important area for future exploration, despite the fact that neither ES nor IPS cells seem to harbor prominent RNPs. It is important to stress that both ES and IPS cells are artificial laboratory products. In vertebrates at least, somatic pluripotency in vivo exists only transiently during the earliest stages of embryonic development. Neoblasts and germ cells on the other hand have evolved as permanently pluripotent cells. Their joint reliance on RNPs could therefore reflect critical accessory functions required for the long-term maintenance of pluripotency. The established role of germ granules and CBs in safeguarding genome integrity (see above) fits this picture. The erasure of epigenetic marks, which remains incomplete in reprogrammed cells (Lister et al. 2011; Ohi et al. 2011), could be a second such possibility worth exploring. Overall, planarian cNeoblasts as naturally occurring pluripotent stem cells ideally complement vertebrate in vitro systems towards the far goal of medically exploiting pluripotency.

## Neoblast heterogeneity

Beyond molecular mechanisms, the distribution of pluripotency within the neoblast population raises the next set of urgent questions. Are all cells expressing *smedwi*-*1*, *PRMT*-*5* or any of the other Neoblast markers pluripotent cNeoblasts? Or are cNeoblasts a minority amongst a majority of transient amplifying progenitors? Expression patterns of Neoblast genes have so far been of little help in this respect. Many Neoblast genes display the typical expression pattern epitomized by *smedwi*-*1* and, importantly, are coexpressed at the single-cell level (Guo et al. 2006; Wagner et al. 2012; Eisenhoffer et al. 2008; Reddien et al. 2005b). Others are likely expressed only in subpopulations of *smedwi*-*1* ^+^ cells (Sakurai et al. 2012; Rossi et al. 2006; Scimone et al. 2010). Further evidence for heterogeneity in terms of morphology and gene expression comes from FACS-sorted radiation sensitive cell populations (Higuchi et al. 2007; Morris et al. 2006; Hayashi et al. 2010; Shibata et al. 2012). Dynamic expression pattern changes during the recovery from near-lethal irradiation have further been interpreted in favor of a more radiation-resistant Neoblast subpopulation in association with the ventral nerve cords (Salvetti et al. 2009). However, the relevance of these observations to Neoblast potency remains tentative, because a conclusive differentiation between stem cells and transient amplifying cells necessitates assessment of cell lineages.

The single Neoblast transplantations by Wagner and colleagues (2011) are landmark achievements also because they introduce this dimension to Neoblast research. Starting from a cell population sorted according to size and complexity ((X1)FS), the authors report seven whole-animal reconstitutions out of 130 single cell transplant attempts (Wagner et al. 2011). The starting population therefore must have contained at least 5% pluripotent cells, whereas the actual proportion might be significantly higher due to the technical challenges involved.

More than 5% pluripotent cells in a label-free sorted fraction lead to the important conclusion that cNeoblasts cannot be as exceedingly rare as, for example, vertebrate haematopoietic stem cells (Lensch and Daley 2004). However, the proportion of cNeoblasts amongst *smedwi*-*1* ^+^ cells and consequently the possible existence of transient amplifying or lineage-restricted populations remain unknown. A further important milestone will be the localization of cNeoblasts within the planarian tissues. Addressing these issues will require a new generation of experiments linking molecular markers with assays of differentiation potential. The recently reported sub-fractionation of living Neoblasts with surface antibodies represents a further step into this direction (Moritz et al. 2012).

## Neoblast homeostasis

The maintenance of appropriate stem cell numbers is a central challenge in all stem cell systems. Excessive stem cell divisions can lead to cancer, a loss of stem cells to a halt of tissue turnover and premature ageing (Arwert et al. 2012). Stem cell homoeostasis has to balance the two foundations of stemness, self renewal and differentiation into multiple progeny (Weissman 2000). Every single stem cell division challenges homeostasis: Symmetric divisions resulting in two stem cells increase stem cell numbers. Symmetric divisions resulting in two differentiating daughters signify a net loss of one stem cell. Only asymmetric divisions (i.e., the production of one stem cell and one differentiating daughter) maintain the status quo. *Drosophila* neuroblasts regulate the fate choice cell intrinsically via the asymmetric segregation of differentiation determinants into their daughter cells (Prehoda 2009). The mammalian gut is an example of a stem cell system operating without intrinsic division asymmetries (van der Flier and Clevers 2009). Such systems can maintain homeostasis at the population level despite stochastic outcomes of individual divisions (González-Estévez et al. 2012b; Simons and Clevers 2011). Practically nothing is known regarding the regulation of cNeoblast divisions at the single cell level. However, the fact that cNeoblasts self-amplify (i.e., divide symmetrically) in addition to producing progeny (i.e., asymmetric divisions) during the recovery from partial irradiation strongly suggests population-level control mechanisms (Wagner et al. 2011, 2012). Further progress along these lines, including questions regarding the niche of cNeoblasts, will have to await their localization within the tissue.

Controlling stem cell division rates constitutes a further critical aspect of organismal stem cell homeostasis. Many systems display a basal rate of stem cell activity during normal tissue turnover, which can dramatically increase in response to injury, for example (reviewed by Arwert et al. 2012). Similarly, the planarian Neoblast system clearly switches between regulatory states. At steady state, Neoblasts divide at a basal rate much like stem cells in high turnover tissues like the gut or the epidermis. Interestingly, neoblast divisions continue during prolonged periods of starvation, even though starving planarians shrink continuously due to decreasing cell numbers (Baguñà and Romero 1981; Oviedo et al. 2003; Takeda et al. 2009; González-Estévez et al. 2012a). The catabolism of dying cells likely fuels divisions during starvation, thus maintaining tissue turnover at the cost of progressive cell loss (Baguñà and Romero 1981; Baguñà et al. 1990). Feeding has the opposite effect: Ingested food elicits a rapid and pronounced increase in the fraction of Neoblasts in M-phase, peaking already 8 h post feeding and taking several days to return to baseline (Baguñà 1974; Kang and Sánchez Alvarado 2009). This transient increase in Neoblast divisions temporarily tips the steady state towards a net increase in cell numbers, leading to a burst of growth at the whole animal level. Wounding has a similar effect on Neoblast mitotic activity, causing a sharp peak of M-phase Neoblasts within 6–8 h post injury and a second, more sustained mitotic peak in the wound vicinity at 3 days post wounding (Wenemoser and Reddien 2010; Baguñà 1976b). Progenitors generated during wounding are thought to migrate to the injury site and to give rise to the regeneration blastema (Aboobaker 2011; Forsthoefel and Newmark 2009; Baguñà 2012). Interestingly, injuries as small as a needle prick can elicit the rapid injury response component (Wenemoser and Reddien 2010).

What constitutes the Neoblast reserve capacity — a non-cycling Neoblast subpopulation set aside specifically for activation during growth or repair? Or acceleration of cell cycle dynamics within a continuously cycling population? The mitotic peak occurring already at 6–8 h post wounding/feeding has been interpreted in favor of a G2-arrested subpopulation (Baguñà 1974; Saló and Baguñà 1984). The introduction of BrdU pulse labeling by Newmark and Sánchez Alvarado challenged this idea (Newmark and Sánchez Alvarado 2000). The authors calculated a mean G2 duration of 5 h based on fractional labeling of mitoses, suggesting that a G2-arrested population might not be required to explain the rapid feeding-induced rise of mitoses. Further, the finding that 12 h after feeding a BrdU/food mixture or after 3 days of continuous BrdU injections all dividing cells were BrdU-positive, led the authors to the conclusion that all Neoblasts divide continuously. A caveat to this interpretation was the intermittently discovered activation of Neoblasts even by injection injuries, which has so far precluded BrdU measurements of neoblast dynamics in the unstimulated state (Wagner et al. 2011; Wenemoser and Reddien 2010). This leaves open the very real possibility that under unstimulated conditions some Neoblasts might take considerably longer than 3 days to divide or maybe even not divide at all. Hence the cellular mechanisms by which the planarian stem cell system adjusts progeny output to changing physiological needs remain unclear. The importance of the question also in terms of Neoblast population heterogeneity and the availability of more versatile Neoblast markers now warrant an experimental return to these questions. The recently developed soaking method for BrdU delivery (Cowles et al. 2012) might offer a first opportunity to examine Neoblast cycling dynamics under unstimulated conditions.

## Signals affecting Neoblast division

The fact that the Neoblast system can dramatically change its progeny output emphasizes the importance of proliferation controlling signals. From the above, the Neoblast division control network can be expected to encompass local self-renewal signals, global activating signals released in response to wounding or changes in metabolic status, as well as negative feedback loops mediating the return to basal division rates. So far, only incidental observations provide glimpses of the molecular underpinning. Early studies suggested an effect of neuropeptides on Neoblast division rates (Baguñà et al. 1989; Saló and Baguñà 1986), which have been interpreted in support of a general role of the nervous system in controlling Neoblast proliferation (Rossi et al. 2012; Baguñà et al. 1989). Hh signaling exerts a global influence on mitotic cell densities and Hh is also expressed in the nervous system (Rink et al. 2009; Yazawa et al. 2009). However, neither in this case nor in others has an explicit role of the nervous system in Neoblast proliferation control been established. Jun-kinase activation appears to be generally required for the initiation of mitosis (Tasaki et al. 2011b), but its upstream regulators remain to be identified. *Smed*-*inx11*, a gap junction gene required for Neoblast maintenance, has been suggested as part of local niche signals (Oviedo and Levin 2007). The two FGF receptors specifically expressed in Neoblasts are further interesting candidates in this respect (Wagner et al. 2012). The Wnt signaling pathway, which provides important niche signals in multiple systems (Nusse 2008; Klaus and Birchmeier 2008), has so far not been implicated in planarian stem cell maintenance. Instead, planarian Wnt signaling has prominent effects on body plan patterning (Gurley et al. 2008; Petersen and Reddien 2008; Iglesias et al. 2008). Feedback loops dampening the Neoblast response may include SMED-EGFR-1, an EGF receptor expressed in the planarian intestine (Fraguas et al. 2011) and *Dj*-*P2X*, a likely ATP-gated plasma membrane channel expressed in a subset of Neoblasts (Sakurai et al. 2012). The TOR pathway, which has diverse functions in cell growth and metabolism (Zoncu et al. 2011), recently emerged as an important component in orchestrating the Neoblast response to wounding and regeneration (Peiris et al. 2012; Tu et al. 2012; González-Estévez et al. 2012b). Neither wound nor food response signals have been identified, but a recent report implicates planarian Insulin signaling in the maintenance of Neoblast divisions during starvation (Miller and Newmark 2012).

Clearly, key elements of the neoblast control network await identification and much remains to be learned about the mechanistic and dynamic interconnections between pathways and cell types. Further, planarians not only regulate Neoblast proliferation, but also the rates of differentiated cell removal, likely even in a tissue-specific manner (González-Estévez et al. 2012a; Pellettieri et al. 2010). The signals and signaling pathways controlling this important part of the steady state equation remain largely unknown, but the recent studies of planarian TOR signaling tentatively implicate this pathway (Peiris et al. 2012; Tu et al. 2012).

## Orchestration of Neoblast differentiation

A further critical factor in maintaining the steady state between stem cells and differentiated cells is the appropriate orchestration of stem cell progeny differentiation. Clearly, the complexity of this task increases with the number of available choices. Vertebrate adult stem cells are tissue specific and possible progeny fates are therefore limited, e.g., Goblet-, Paneth-, Enteroendocrine cell and absorptive enterocytes in the vertebrate intestine (van der Flier and Clevers 2009). Pluripotent cNeoblasts giving rise to all planarian cell types represent a worst case scenario in terms of fate choice complexity, raising a number of important questions: Are Neoblast progenitor fate choices intrinsically programmed or influenced by the momentary needs of the tissue? At which point of the lineage downstream of cNeoblasts are cell fates determined? How are fate choices coordinated with the global organization of the planarian body plan?

The so-called category markers discovered by Eisenhoffer and colleagues provided first insights into the spatiotemporal orchestration of Neoblast progeny differentiation (Eisenhoffer et al. 2008). Based on the differential downregulation kinetics of irradiation sensitive genes and on BrdU pulse labeling, this study defined two gene categories likely expressed sequentially in differentiating postmitotic Neoblast progeny (Cat. 2: Early progeny; Cat. 3: Late progeny; Cat. 1: dividing Neoblasts). The distinct expression domains of the category markers suggested that Neoblast progeny are “born” deep within the mesenchyme and undergo outward migration during the course of differentiation (Eisenhoffer et al. 2008), consistent with previous BrdU-pulse labeling studies (Newmark and Sánchez Alvarado 2000). The Category markers also provided an important assay for identifying molecular regulators of progeny differentiation. Pearson and colleagues found that *Smed*-*p53*, the homologue of the vertebrate tumor suppressor p53, is required for the transition between Cat. 1 and 2 expression, as *Smed*-*p53*(*RNAi*) causes the accumulation of mitotic Neoblasts at the expense of progenitors (Pearson and Sánchez Alvarado 2010). Erk kinase activation appears to be similarly required for exiting the proliferative state (Tasaki et al. 2011a). The formation of Cat. 3 expressing cells, on the other hand, requires the polycomb chromatin remodeling complex *Smed*-*CHD*-*4* (Scimone et al. 2010; Wagner et al. 2012). A further chromatin remodeling complex member, *Dj*-*RbAp48*, has also been implicated in Neoblast differentiation, emphasizing the likely importance of chromatin rearrangements during the transition from pluripotency to terminal differentiation (Bonuccelli et al. 2010). Recently, Wagner and colleagues found that the relative ratios between Cat. 1, -2, and -3 expressing cells remain constant during the clonal expansion of cNeoblast colonies in sublethally irradiated animals (Wagner et al. 2012). Besides the interesting conclusion that self-amplifying Neoblast divisions occur at a fixed proportion to progeny-generating divisions (at least during clonal expansion), this finding allowed a quantitative distinction between Neoblast self-renewal and differentiation defects. This new tool confirmed the role of previously reported differentiation factors and further implicated the likely chromatoid body component *Smed*-*Vasa*-*1* and an as yet uncharacterized zinc finger transcription factor into regulating the transition between proliferating Neoblasts and postmitotic progeny. What remains unclear is the identity of the cells expressing the category markers, in particular the extent by which they represent generic differentiation stages or early stages of a specific lineage with unknown end point (Eisenhoffer et al. 2008; Scimone et al. 2010).

One recent publication in particular provides a fascinating bird’s eye view of the differentiation process from Neoblasts all the way to the terminally differentiated cells types of the planarian eye (Lapan and Reddien 2011). The authors defined a set of transcription factors expressed in- and required for the differentiation of planarian optic cup cells and photoreceptive neurons. Using these tools to ask where and when eye precursors arise during head regeneration, the authors reached two important conclusions: First, eye precursors differentiate far from the forming eye, giving rise to a ~200 μm long “trail” of eye progenitor cells migrating distally from the base of the blastema. Second, at its proximal boundary (i.e., close to the old tissue), the optic cup precursor cells were found to co-express the Neoblast marker genes *Smed*-*H2b* and *smedwi*-*1*. Similarly, transcription factors required for protonephridial cell fate specification (Scimone et al. 2011), general neuronal differentiation (Wenemoser et al. 2012), tyrosine hydroxylase as marker of dopaminergic neurons (Nishimura et al. 2011) and a muscle-specific myosin heavy chain (Hayashi et al. 2010) have been shown to co-express with Neoblast markers, generalizing the onset of differentiation towards specific cell fates already within *smedwi*-*1* expressing cells.

## The plight of pluripotency: conceptual problems of lineage organization

**Fig. 3:**
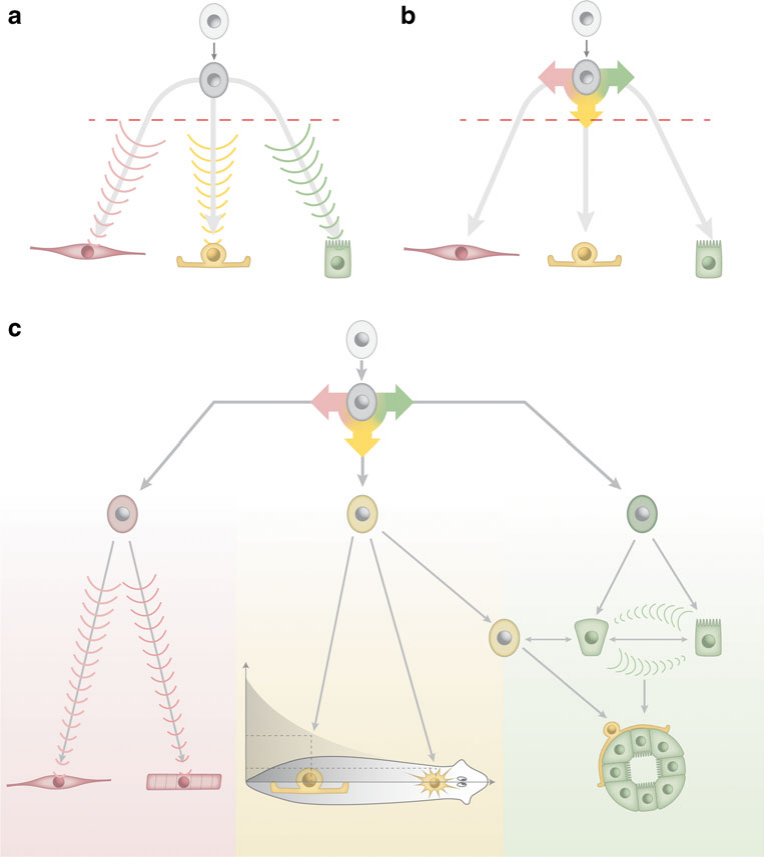
Concepts in stem cell lineage organization. **a** “Differentiation on demand”: signals from differentiated cells instruct the fate choice of a pluripotent progenitor. **b** Stochastic differentiation. Purely cell-intrinsic mechanisms determine the fate choice of a pluripotent progenitor. Tissue loss due to injury (*red line* symbolizing amputation plane) could affect fate choices in **a**, but not in **b**. **c** Hypothetical differentiation hierarchy utilizing a combination of mechanisms. Initial lineage segregation occurs via cell intrinsic mechanisms (*top*). Terminal cell fates arise from additional choice points within lineages, involving a combination of (1) local “differentiation on demand” signals in the tissue environment (*bottom left*); (2) global patterning signals (centre) and (3) self-assembly of complex structures by means of spatiotemporal interactions amongst progenitors. Note that the scheme does not distinguish neoblasts and postmitotic progeny: initial lineage segregation (*top*) amongst postmitotic progeny would imply uniform neoblast pluripotency; lineage segregation already within dividing cells would imply neoblast heterogeneity in form of transit amplifying cells

The mechanisms and conceptual principles orchestrating progenitor differentiation in planarians are currently mainly a matter of speculation. Are progenitor fate choices under the control of external signals, such that particular cell types could differentiate “on demand” (Fig. 3a)? Or are fate choices the outcome of stochastic cell intrinsic decision making processes, as for example the differentiation of retinal precursor cells (Gomes et al. 2011)? “On demand” models entail flexibility in matching progenitor differentiation to local needs, for example the replacement of a dying cell by means of a concomitantly released differentiation cue. However, such models necessitate a multitude of specific “replace me” signals for the multitude of cell types in the organism and, more significantly, would render regeneration impossible as soon as all cells of a particular type were lost (e.g., loss of photoreceptors and their differentiation signal in a head amputation). The fact that planarians can regenerate all tissues and organs de novo therefore challenges any model requiring the presence of a differentiated cell for progenitor differentiation into this cell type. By contrast, progenitor fate choice via cell intrinsic mechanisms could accomplish the de novo specification of lost cell types, yet again at conceptual costs. During regeneration, the provision of the exact numbers and types of progenitors long before and far away from their assembly into tissues and organs (Lapan and Reddien 2011) is difficult to envisage on basis of stochastic mechanisms. During steady state, purely cell intrinsic fate choice would result in the exact same progenitor production throughout the animal, entailing the differentiation of cell types in places where they are of little use (e.g., photoreceptors in the tail). Apoptosis or long-range migration could conceivably deal with supernumerary or out-of-place progenitors, but further phenomena difficult to envisage on basis of purely cell-intrinsic fate choice mechanisms include the size-threshold-dependent differentiation of reproductive organs (Newmark et al. 2008) or the changing need for epithelial cells during growth and degrowth due to changing surface to volume ratios.

Both the above scenarios run into conceptual difficulties because they assume terminal cell fate specification at a single control point. However, a single control point is unlikely already in face of the sheer multitude of planarian cell types existing. Furthermore, cell fate choices during embryonic development result from the gradual restriction of differentiation potential via multiple differentiation cues. A hierarchical organization of Neoblast differentiation therefore appears likely (Baguñà et al. 1990), in particular one incorporating relatively general lineage restrictions at the top of the lineage tree (e.g., “neuronal”, “intestine” and “muscle”, or possibly “ectoderm”, “endoderm”, “mesoderm”) and the gradual specification of terminal cell fate (e.g., precise neuronal subtype) at later differentiation stages (Fig. 3c). The observed expression of the pigment cup marker *Smed*-*tyrosinase* in the immediate vicinity of the regenerating eye, yet the expression of “early” eye markers in much wider domains is broadly consistent with such a model (Lapan and Reddien 2011). Significantly, cell fate decisions in differentiation hierarchies are not taken at once, but represent the cumulative outcome of multiple intermediate control points. Differentiation hierarchies can therefore incorporate both cell-intrinsic and signal-mediated fate choice mechanisms at different control points, thus combining stability with flexibility in the fate choice problem. Execution of the initial lineage choice by cell intrinsic mechanisms, as for example the stochastic cell fate choice of retinal progenitors (Gomes et al. 2011), provides an appealing mechanism for guaranteeing long-term stability of the lineage tree. The aforementioned caveat of generating out of place progenitors becomes less important the more general the initial lineage choices are. Except for the brain and pharynx, all major planarian organs including the CNS are rather uniformly distributed, suggesting similar requirements for generic “neuron”, “muscle” or “intestine” progenitors throughout the planarian body plan. Intrinsic choice mechanisms could remain in place during regeneration, as the de novo formation of any tissue would again require the same generic progenitor classes.

On the other hand, signal-mediated “on demand” progenitor fate choice mechanisms are appealing towards later stages of the differentiation hierarchy, especially because they leave room for self-organization. The de novo regeneration of the planarian eye via the coalescence of individually migrating progenitors arising far from their target site provides a striking example of self-organization (Lapan and Reddien 2011). Assembling a structure as complex as the eye necessitates both multiple cell fate choices and precise spatial arrangements of cells. Pattern formation, the analysis of collective properties arising from dynamic interactions (Bois et al. 2011; Gierer and Meinhardt 1972; Howard et al. 2011), provides an appealing conceptual framework for rationalizing de novo organ regeneration. Accordingly, a general commitment to a particular lineage could initiate specific signaling interactions amongst similar progenitors and the tissue environment, leading to progenitor coalescence concomitant with terminal progenitor fate choices (Fig. 3c). Pattern formation not only as consequence, but also as cause of progenitor fate choice could alleviate the aforementioned need for precise “counting” of progenitors long before the distant assembly of the organ. Nevertheless, it is important to stress the conceptual nature of the above discussion. The possibility remains that final fate choices occur already high up in the lineage and that Neoblasts in the postblastema region are therefore able to precisely gauge future progenitor requirements after all. In fact, the co-expression of the dopaminergic neuron marker *Dj*-*tyrosine hydroxylase* with *Dj*-*piwi*-*1* (Nishimura et al. 2011), and the apparent specification of pigment cup cells at a similar state (Lapan and Reddien 2011) provide some evidence to this effect. Important questions include the possibility of hierarchical differentiation stages already within the *piwi* ^+^ Neoblast population, the extent by which individual cell types and lineages compete for progenitors and whether regeneration and steady state turnover rely on the same or different lineage choice mechanisms. The observation that *Smed*-*runt1*(*RNAi*) leads to eye defects in regenerating, but not in uninjured animals (Wenemoser et al. 2012), could point towards regeneration specific fate choice mechanisms.

## Progenitor fate choices and global patterning

Which leaves the crucial question of how the regionalization of the planarian body plan is achieved, that is, why eyes and their associated cell types form only in the head and not in the tail of the animals? Or how regeneration rebuilds exactly what is missing. Clearly, these phenomena necessitate an influence of global body plan patterning signals over local cell fate choices (Lobo et al. 2012; Reddien 2011; Forsthoefel and Newmark 2009; Baguñà 2012). Mechanistic detail is once again lacking, but the profound effects of altered *Smed*-β-*catenin*-*1* activity are conceptually important. β-Catenin is the intracellular effector of canonical Wnt signals (Clevers and Nusse 2012). In planarians, decreased activity of β-*catenin*-*1* forces head regeneration irrespective of the wound context (Gurley et al. 2008; Iglesias et al. 2008; Petersen and Reddien 2008), increased activity has the opposite effect by dominantly forcing tail regeneration (Gurley et al. 2008). These phenotypes are remarkable, since the identity switch of regenerating tissues resulted from global manipulations of a single gene. β-*Catenin*-*1* therefore cannot act as simple cell intrinsic fate determinant, which would have turned RNAi-treated animals into uniform masses of either anterior or posterior marker expressing cells. Instead, the result of perfectly patterned heads and tails indicates that β-*catenin*-*1* activity exerts a binary choice high up in a tissue fate program. Embryonic organizers provide an interesting paradigm for how this might occur. Organizers are self-organizing and self-maintaining signaling centers (incidentally often specified via β-catenin), that orchestrate embryonic axis establishment (De Robertis 2009). Two aspects of the organizer concept are particularly appealing in planarian regeneration. First, they self-assemble on basis of dynamic interactions between cells, which can account for pervasive head or tail regeneration competence without the need for pre-localized determinants (Meinhardt 2008, 2009). Second, organizers determine and spatially organize the lineage choices of surrounding cells (Niehrs 2004). The role of *Smed*-β-*catenin*-*1* could consequently signify the choice between establishing one of two self-maintaining signaling systems at the injury site — either a Wnt/β-*catenin*-*1*-dependent positive feedback system mediating tail regeneration, or a mutually exclusive Wnt inhibitory system at sites of head regeneration. The signaling environments established by such “blastema organizers” could subsequently orchestrate lineage choices and spatial arrangement of differentiating progeny towards the reconstruction of a head or tail, respectively.

An influence of patterning signals over lineage choices necessitates region-specific cell fate determinants or factors mediating regional differentiation competence. An example for the former could be the LIM-homeodomain transcription factor *Dj*-*ISLET*. This protein is specifically required for proper tail regeneration, expressed in a narrow stripe of differentiating progeny and, interestingly, required for the maintained expression of tail fate determining Wnt ligands (Hayashi et al. 2011). The TALE-homeobox gene *Smed*-*prep* on the other hand is broadly required for head induction downstream of *beta*-*catenin*-*1*(*RNAi*) (Felix and Aboobaker 2010). TALE-homeobox genes can function as Hox co-factors, which would fit with a role of *Smed*-*prep* as head region selector gene. Importantly, the mechanisms specifying blastema fate also participate in the maintenance of the planarian body plan at steady state. *Smed*-*betaCatenin1*(*RNAi*) in non-regenerating animals causes the conversion of the tail into a head and the emergence of ectopic heads all along the body edge (Gurley et al. 2008; Iglesias et al. 2008; Petersen and Reddien 2008). Gene expression patterns suggest permanently high canonical Wnt signaling in the tail and low levels in the head, as in the respective regeneration blastemas (Gurley et al. 2010; Petersen and Reddien 2009; Adell et al. 2009). β-Catenin and canonical Wnt signaling therefore exert the same high-level lineage choices at steady state as during regeneration and the head-territory specific expression of *Smed*-*prep* in non-regenerating animals indicates the participation of similar factors. The challenge over the coming years will be the cell biological dissection of the signaling cascades and their influence on gene regulatory networks.

Both the regionalization of the planarian body plan and its astonishing regenerative abilities are therefore likely two different sides of the same coin: a pluripotent differentiation hierarchy under the influence of a regulative patterning system. The uniquely dynamic body architecture of planarians arises from the intimate entwinement between the stem cell lineage and the patterning systems. Progenitors differentiate according to patterning signals into cells that produce patterning signals. The end of cell fate choices therefore once again becomes a beginning and the pattern continuously replaces itself. How such pattern of signals manifests itself in the physical shape and proportions of the planarian body plan is possibly the most fascinating challenge posed by the model system. Meeting it will require a multidisciplinary systems biology approach, combining cell biology, biophysics and mathematical modeling. The prize are fundamental insights into a universal design principle of biological systems, the dynamic steady state.

## Zooming out: stem cells, pluripotency and evolution

Finally, how unusual are planarians and their pluripotent adult stem cell system in the context of evolution? Interestingly, Neoblast-like somatic stem cells are being discovered in a growing list of animals. *piwi* ^+^, *bruno* ^+^ and *PDX10* ^+^ positive stem cells occur within the posterior growth zone of several annelid species, which adds new segments during growth (Giani et al. 2011; Yoshida-Noro and Tochinai 2009; Rebscher et al. 2007). Acoels, either basal bilateria or basal deuterostomes (Ruiz-Trillo 1999; Philippe et al. 2011; Bourlat et al. 2006), harbor a Neoblast system that closely resembles the one of planarians (De Mulder et al. 2009). Ctenophores (Alié et al. 2011) and cnidarians maintain dynamic stem cell systems that express *piwi*, *vasa* and *nanos* and, at least in the case of the cnidarians, are able to regenerate the germ cells as well (Mochizuki et al. 2001; Rebscher et al. 2008; Denker et al. 2008; Mochizuki et al. 2000). Sponges as the evolutionarily oldest extant metazoans harbor pluripotent *piwi*+ stem cells that also give rise to the germ line (Funayama et al. 2010; Funayama 2010). The tunicates, a subphylum at the base of the vertebrates, provide fascinating examples of whole body regeneration from pluripotent and *piwi*+ stem cells (reviewed by Kürn et al. 2011).

Flatworms are therefore neither unique in possessing pluripotent somatic stem cells, nor are they the only animals that undergo continuous turnover. Rather, stem cell systems across the animal kingdom come as a continuum of potencies and activities, in which Planarians mark a dynamic extreme. Hydra is situated nearby, displaying continuous body turnover, yet driven by independent multi- and unipotent cell lineages. Vertebrates with extremely tissue-specific turnover rates and tissue-specific stem cells reside somewhere in the middle, while *C*. *elegans* completely lacking adult somatic stem cells and somatic cell turnover occupies the “static” extreme of the spectrum. Similarly, the mechanisms segregating the pluripotent germ line from somatic lineages range from differentiation from pluripotent somatic stem cells in hydra, planarians or sponges via embryonic epigenesis in mouse to the segregation of maternally inherited “immortal” germ plasm in fly and *C*. *elegans* (Extavour 2007). Based on these observations, pluripotent somatic stem cells have been suggested as the ancestral state of animal stem cell systems (Agata et al. 2006; Extavour 2007; Blackstone and Jasker 2003). Tissue specific somatic stem cells might represent a secondary adaptation to the increasing size and functional specialization of body parts. This view would predict that the molecular components of pluripotency were initially shared between the pluripotent somatic stem cells and the germ line, before becoming increasingly restricted to the germ line. The expression of *piwi* and other germ line genes in the pluri- and multipotent stem cell systems of invertebrates, but generally not in the lineage restricted vertebrate stem cells, would indeed fit this picture. Or are cNeoblasts and other pluripotent invertebrate stem cells pre-meiotic germ cells instead that have maintained the ability to contribute to the somatic lineages? Clearly, studying the embryonic origins of Neoblasts and similar cells in other emerging invertebrate model systems now provide experimental opportunities to approach the evolution of pluripotency and animal stem cell systems in general.

## Summary and outlook

Planarians as model system contribute unique perspectives to current problems in stem cell research.

Their first asset is an abundance of adult stem cells, so called cNeoblasts. The recent demonstration of cNeoblast pluripotency in single cell transplants has been an important achievement (Wagner et al. 2011). The phylogenetic distances between cNeoblasts and mammalian ES and IPS cells provide unique opportunities for investigating whether pluripotency emerges from an evolutionarily conserved core mechanism or instead from general interactions between cellular processes as a systems property. The unknown degree of population heterogeneity amongst Neoblasts remains both a conceptual and experimental bottleneck. New tools are required for the definition of Neoblast subpopulations based on molecular and functional criteria (e.g., stem cell potency, self-renewing capacity). Establishing the fraction of pluripotent cNeoblasts amongst *smedwi*-*1* expressing cells, the isolation of pure cNeoblast preparations, the establishment of *in vitro* culture conditions and the localization of cNeoblasts within their tissue microenvironment represent important future milestones.

Second, Neoblasts are the sole source of new cells in planarians and they divide continuously to replace all differentiated cell types. Planarians therefore exist in a dynamic steady state between a single proliferating stem cell type and multiple short-lived differentiated cells. Maintenance of the steady state constitutes a universal problem in any stem cell containing tissue, yet the phenomenon remains poorly understood. Their complete turnover within a matter of weeks makes planarians uniquely suitable for studying the underlying mechanisms and conceptual principles. Key challenges include the identification of signaling systems controlling Neoblast proliferation and differentiated cell turnover, the orchestration of progenitor differentiation downstream of the pluripotent cNeoblasts and a systematic analysis of cell movements at steady state and during regeneration. The development of planarian transgenesis tools are especially important in addressing these questions.

Third, the ability of planarians to maintain their body plan in face of constant turnover is simply fascinating. Planarians grow and de-grow by changing overall cell numbers depending on food availability, leading to a 40-fold variation in body size between 0.5 mm and 20 mm in the case of *S*. *mediterranea*. Moreover, even random injury remnants can restore the body plan by regeneration. Understanding the mechanisms that maintain and re-establish the dynamic steady state between Neoblasts and differentiated cells within the exact shape, size and proportions of the planarian body plan constitutes possibly the greatest frontier in planarian stem cell research. A crucial missing link between morphogenesis and the stem cell system is the currently unknown intersection point of patterning signals with progenitor fate choices. The origin and mechanisms of the mechanical forces required to convert signaling patterns into physical shape represents a further frontier. As for any steady state, understanding the planarian body plan in mechanistic detail will ultimately require mathematical modeling. Quantitative measurements are a crucial prerequisite to move into this direction and data relating to such parameters as progenitor production rates, cell flows and cell turnover rates will represent important contributions.

What planarians can uniquely contribute to our understanding of stem cells is the systems perspective: an integrated view of the stem cell and its descendants as a dynamic steady state.
